# Molecular Dynamics of the Neuronal EF-Hand Ca^2+^-Sensor Caldendrin

**DOI:** 10.1371/journal.pone.0103186

**Published:** 2014-07-24

**Authors:** Pasham Parameshwar Reddy, Vijeta Raghuram, Johannes Hradsky, Christina Spilker, Asima Chakraborty, Yogendra Sharma, Marina Mikhaylova, Michael R. Kreutz

**Affiliations:** 1 RG Neuroplasticity, Leibniz Institute for Neurobiology, Magdeburg, Germany; 2 Centre for Cellular and Molecular Biology, CSIR, Hyderabad, India; 3 Cell Biology, Faculty of Science, Utrecht University, Utrecht, The Netherlands; University of Oldenburg, Germany

## Abstract

Caldendrin, L- and S-CaBP1 are CaM-like Ca^2+^-sensors with different N-termini that arise from alternative splicing of the Caldendrin/CaBP1 gene and that appear to play an important role in neuronal Ca^2+^-signaling. In this paper we show that Caldendrin is abundantly present in brain while the shorter splice isoforms L- and S-CaBP1 are not detectable at the protein level. Caldendrin binds both Ca^2+^ and Mg^2+^ with a global K_d_ in the low µM range. Interestingly, the Mg^2+^-binding affinity is clearly higher than in S-CaBP1, suggesting that the extended N-terminus might influence Mg^2+^-binding of the first EF-hand. Further evidence for intra- and intermolecular interactions of Caldendrin came from gel-filtration, surface plasmon resonance, dynamic light scattering and FRET assays. Surprisingly, Caldendrin exhibits very little change in surface hydrophobicity and secondary as well as tertiary structure upon Ca^2+^-binding to Mg^2+^-saturated protein. Complex inter- and intramolecular interactions that are regulated by Ca^2+^-binding, high Mg^2+^- and low Ca^2+^-binding affinity, a rigid first EF-hand domain and little conformational change upon titration with Ca^2+^ of Mg^2+^-liganted protein suggest different modes of binding to target interactions as compared to classical neuronal Ca^2+^-sensors.

## Introduction

EF-hand-type Calmodulin (CaM) like Ca^2+^-sensor proteins play an important role in neuronal Ca^2+^-signaling [1], [2]. Two families have been identified that are prominently expressed in brain, the Neuronal Calcium Sensor (NCS) and the neuronal Calcium Binding Protein (nCaBP) family [2]. The nCaBP family, comprising Caldendrin/CaBP 1–5 and Calneuron-1 and -2 (also called CaBP8 and 7) arose much later during vertebrate evolution [2], [3], [4]. With respect to their EF-hands they exhibit greater similarity to CaM than members of the NCS family. It is therefore believed that nCaBPs evolved directly from the ancestral CaM [2], [3], [4].

Caldendrin is the first identified member of the nCaBP family [5]. Alternative splicing of the Caldendrin/CaBP1 gene generates three isoforms [6] (Figure S1A) from which only Caldendrin is abundant in brain [7], [8], although conflicting results regarding this issue have been reported [9]–[11]. Caldendrin has in comparison to other EF-hand Ca^2+^-sensors a number of unique properties. It lacks a N-myristoylation sequence, which is present in many NCS proteins and in the shorter splice isoforms L- and S-CaBP1 [2] (Figure S1A). The extended N-terminus of Caldendrin leads to a bipartite structure with a highly basic and proline-rich N-terminal and an acidic C-terminal part [5]. Exon 1 is 17,38 kb upstream of the exons encoding the common C-terminus [7] (Figure S1A). Interestingly, Caldendrin can be found to a large degree in the Triton-X100 insoluble fraction and tightly associates with the postsynaptic density in subcellular fractionation experiments [5], [7], [12]. In addition, Caldendrin has been implicated in neuropsychiatric disorders [13]–[15].

In contrast to S-CaBP1, very little is known about the molecular characteristics, structure and ion binding properties of Caldendrin. The four common EF-hands in L−/S-CaBP1 and Caldendrin are organized into an N- and C-terminal lobe [16]–[18] (Figure S1A). EF-hand 1 constitutively binds Mg^2+^, whereas EF-hand 3 and 4 of the C-terminal lobe are high affinity sites for Ca^2+^ binding [19] (Figure S1A). EF-hand 2 is cryptic and does not bind either Mg^2+^ or Ca^2+^ [16]–[19]. The C-terminal domain comprising EF-hands 3 and 4 shows the classical close to open conformational change upon Ca^2+^-binding in S-CaBP1 [16]–[18].

The basic N-terminal half of Caldendrin is unique to the protein and the consequences for the structure, possible interaction with the acidic C-terminal region and the impact on Ca^2+^-binding are currently unknown. Studies on the interaction of S-CaBP1 and Caldendrin with the L-type voltage-gated calcium channel (VGCC), Cav1.2, have shown that S-CaBP1 and Caldendrin interact with the channel using different molecular determinants and that both proteins have different effects on channel activity [11]. In the present work we investigated biophysical properties, self-association, ion binding and resulting changes in surface hydrophibicity of Caldendrin in comparison to the shorter isoforms.

## Materials and Methods

### Ethics Statement

In the present experiments, animal care and procedures were approved and conducted under established standards of the German federal state of Sachsen-Anhalt (Institutional Animal Care and Use Committee: Landesverwaltungsamt Sachen-Anhalt; License No. 42502-2-987IfN), Germany in accordance with the European Communities Council Directive (86/609/EEC).

### cDNA constructs and site directed mutagenesis

All basic cloning steps were performed according to standard protocols of molecular biology and molecular cloning. Caldendrin, Caldendrin-N-terminus (Caldendrin-Nt, residues 1–136), Caldendrin-C-terminus (Caldendrin-Ct, residues 137–298), L- and S-CaBP1 proteins were cloned into the pMXB10 vector (‘Impact™ system’, New England Biolabs, Frankfurt am Main, Germany) replacing MBP. Newly generated constructs were verified by sequencing analysis. A trptophane (Trp) residue was introduced in place of a phenylalanine (Phe) in each of the EF1W and EF3W mutants. Mutations were introduced by Pfu PCR using mutagenic oligonucleotides following the QuikChange II site-directed mutagenesis kit protocol (Stratagene, La Jolla, CA).

### Preparation of rat and mouse brain and retina lysates and immunoblotting

Sprague Dawley rats from the animal facilities of the Leibniz-Institute were anesthetized with isofluorane and then killed by decapitation. Freshly dissected adult rat tissue was snap frozen in liquid nitrogen and stored at –80°C. Homogenization was done in TBS containing protease inhibitor cocktail (Complete, Roche Diagnostics Mannheim, Germany) using a homogenizer (Potter Elvehjem tissue grinder) in a ratio of 10 ml/g wet weight. Tissue homogenates were then mixed with 4x SDS sample buffer (250 mM Tris pH 6.8, 8% (v/v) SDS, 40% (v/v) glycerol, 5% (v/v) β-mercaptoethanol, 0.01% (v/v) bromphenol blue) in a ratio of 2∶1, boiled for 10 min and centrifuged at 17000×g for 5 min. Total protein concentration was determined by a amidoblack assay and tissue extracts were then subjected to SDS-PAGE according to standard protocols. Rabbit Caldendrin antisera directed against a full-length fusion protein were generated by EXBIO Diagnostics (Czech Republic). Standard protein purification procedures were used to purify anti-Caldendrin specific antibodies [20], [21].

### Intein purification system

Caldendrin full length, Caldendrin-Nt, Caldendrin-Ct, L- and S-CaBP1 proteins were purified via Intein system (‘Impact™ system’, New England Biolabs, Frankfurt am Main, Germany) according to the manufacturer’s protocol. Briefly, overnight grown primary cultures (*E.coli* BL21 DE3) were inoculated (1% v/v) into secondary cultures and induced at an OD_600_ of 0.6 with 1 mM IPTG. L- and S-CaBP1 were co-expressed with N-myristoyltransferase according to published procedures [22], [23]. After 3–4 hrs induction at 30°C cells were harvested and cell pellets were dissolved in 1x Intein buffer (20 mM Tris, 500 mM NaCl and pH 8.5) containing 1% (v/v) Triton X-100 with protease inhibitor cocktail (Complete, Roche). Cell lysis was done with prolonged sonication (15 min). Centrifugation was done at 10,000 rpm for 30 min, the supernatant was collected and passed through a pre-equilibrated Chitin sepharose (NEB) column equilibrated with 1x Intein buffer. Proteins were eluted by incubation with elution buffer (1x Intein buffer containing 20 mM DDT) over 12 hrs at 4°C. Fractions of sufficient purity (>95%) were pooled together and concentrated using Millipore centrifugal filter with a cut-off range >10 kDa (Merck Millipore, MA, USA). To obtain calcium binding proteins in an apo state for biophysical studies the purified proteins were incubated with 100 µM EDTA and subjected to repeated dilutions and concentrations (up to 8 times) in Millipore centrifugal filters at 4,500×g with Chelex-100 resin (Bio-Rad) treated buffer (50 mM Tris PH 7.4; 100 mM KCl). All measurements utilizing purified proteins were repeated at least three times.

### Extrinsic fluorescence spectroscopy

8-Anilinonaphthalene-1-sulfonate (ANS) fluorescence was used to measure the surface hydrophobicity of Caldendrin and its shorter splice isoforms. The ANS solution (10 mM) was prepared in 100% methanol. 10 µl of this solution was added to the protein sample and incubated for 10 min before recording the spectrum. ANS fluorescence was recorded on a Hitachi F-7000 fluorescence spectrophotometer. Excitation was done at 370 nm and spectra were recorded at wavelengths between 400–600 nm. All spectra were recorded at room temperature in corrected spectra mode using an excitation and emission band pass of 5 nm and 10 nm respectively. The response time was set to 2 sec with a scan speed 100 nm/min to 240 nm/min. Changes in fluorescence spectra were monitored with titration of Mg^2+^ (1 mM), Ca^2+^ (50 µM) and Mg^2+^ (1 mM)+Ca^2+^ (50 µM) (saturation was observed with the given ion concentrations). The respective blank spectra were subtracted from individual spectra.

### Circular dichroism spectroscopy

Circular dichroism (CD) spectroscopy was performed on a Jasco-715 spectropolarimeter. Near-UV CD spectra were recorded at room temperature between 250–340 nm using a quartz cuvette of 0.5 cm path length with a chelex-treated protein sample at a concentration of 10–11 mg/ml. Far-UV CD spectra were recorded at room temperature between 195–250 nm using quartz sandwich cuvettes of 0.1 cm path length with a protein sample at a concentration of 0.1–0.2 mg/ml. The common C-terminus of Caldendrin/CaBP1 was dissolved in 50 mM Tris-HCl pH 7.5, 150 mM NaCl, whereas Caldendrin was kept in 20 mM Tris-HCl pH 8.5, 500 mM NaCl and 5 mM TCEP, to ease solubility of the full length protein at the high concentrations used in the near-UV CD experiments. Each spectrum was obtained from 4 accumulations. 0.5 nm data pitch, 50 nm/min scan speed and 0.5 s response time were selected for the recordings. The working concentrations of ligands used were as follows: Mg^2+^- 5 mM (near-UV), 1 mM (far UV), Ca^2+^- 5 mM (near UV), 100 µM (far UV).

### Chemical unfolding

Chemical equilibrium unfolding of full length Caldendrin under ligand-free (apo) and various ligand-bound conditions was monitored by far-UV CD spectroscopy. For each set, 35 samples were made, each containing the protein at 0.75 mg/ml concentration in 20 mM Tris-HCl pH 8.5, 500 mM NaCl and 5 mM TCEP and an increasing concentration of guanidinium chloride (GdmCl), ranging from 0–6 M with an average increment of 0.17 M/sample. Each set differed in its ligand condition, their working concentration being 5 mM MgCl_2_+1 mM EGTA (Mg^2+^-Caldendrin), 5 mM MgCl_2_+1 mM CaCl_2_ (Mg^2+^Ca^2+^-Caldendrin), 1 mM CaCl_2_ (Ca^2+^-Caldendrin) or nil (Apo-Caldendrin). Ellipticity at 220 nm for each of the 35 samples in each set was plotted against GdmCl. The plots were fit using the ‘two state model of unfolding’ described by the equation:
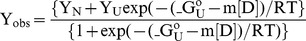
(Where, Y_N_ = ellipticity of the native state; Y_U_ = ellipticity of the unfolded state; ΔG_U_° = standard free energy change of unfolding; [D] = concentration of the denaturant; m = slope of the linear relationship between ΔG_U_° and [D]). The ΔG_U_° and D_½_ ([D] at which 50% of protein was unfolded) obtained from the fit was compared between different groups and conclusions were drawn.

### Isothermal titration calorimery (ITC)

Macroscopic Mg^2+^ and Ca^2+^ binding affinities for Caldendrin and the common Caldendrin/CaBP1 C-terminus were measured using an isothermal titration calorimeter (VP-ITC, Microcal) as decribed previously [22]. For Mg^2+^ titration of apo-Caldendrin, 46 µM of Ca^2+^- free Caldendrin in chelex treated buffer containing 50 mM Tris-HCl pH 7.5 and 100 mM KCl was loaded into the cell and 10 mM of MgCl_2_ prepared in the same buffer was injected. For Ca^2+^ titration of Mg^2+^-bound Caldendrin, 43 µM of Ca^2+^-free Caldendrin in chelex treated buffer containing 50 mM Tris^-^HCl pH 7.5, 100 mM KCl and 5 mM MgCl_2_ was loaded into the sample cell and 5 mM of CaCl_2_ prepared in the same buffer was injected. For Mg^2+^ titration of the common apo-Caldendrin/CaBP1 C-terminus 173 µM of Ca^2+^- free protein in chelex treated buffer containing 50 mM Tris-HCl pH 7.5 and 100 mM KCl was loaded and 15 mM of MgCl_2_ prepared in the same buffer was injected. Ca^2+^ titration of Mg^2+^-saturated protein was done with 120 µM of the common C-terminus in chelex treated buffer containing 50 mM Tris^-^HCl pH 7.5, 100 mM KCl and 5 mM MgCl_2_ and 5 mM of CaCl_2_ prepared in the same buffer was injected. Isotherms were fitted using different models of non-linear curve fitting like one set of sites, two sets of independent sites or sequentially binding sites using the Origin software (version 7) supplied by Microcal. The values for the number of sites (n), association constants (K_a_), enthalpy change (ΔH) and entropy change (ΔS) for each set of sites obtained from the best fit were accepted. Each experiment was repeated at least three times and the average values are shown in Table 1.

**Table 1:** N – Number of binding sites, ΔH – Change in enthalpy, ΔS – Change in entropy, K_a_ – Association constant, K_d_ – Dissociation constant, K_D_ – Global dissociation constant.

Experiment	Model	N	ΔH(cal/mol)	ΔS(cal/mol/K)	Ka(M-1)	Kd(M)	KD(µM)
** Mg^2+^** ** vs** ** apo CDD**	Sequentialbinding	(i) 1(ii) 1	(i) 574.0±25.0(ii) 2061.0±102	(i) 27.6(ii) 24.4	(i) 4.08 E5±1.5 E5(ii) 6.70 E3±6.7 E2	(i) 2.45 E-6(ii) 1.49 E-4	19.1
	One set of sites	(i)	(i) 4165±776.6	(i) 92.5	(i) 1,26E4±2.92E3	(i) 7.9 E-5	79.3
** Ca^2+^** ** vs** ** Mg2+-bound CDD**	Sequentialbinding	(i) 1(ii) 1(iii) 1	(i) −1812±45.9(ii) −3273±54.2(iii) −3409±88.8	(i) 17.3(ii) 11.1(iii) 5.02	(i) 1.31 E5±7.4 E3(ii) 6.66 E4±3.1 E3(iii) 3.98 E3±1.4 E2	(i) 7.6 E-6(ii) 1.5 E-5(iii) 2.5 E-4	}10.7
** Mg^2+^** ** vs** ** apo C-CDD**	One set of sites	(i) 2±0	(i) −950.0±0	(i) 13.5	(i) 4.4 E3±184	(i) 2.27 E-4	227
** Ca^2+^** ** vs** ** apo C-CDD**	Two sets of sites	(i) 1.73±0.102(ii) 0.841±0.169	(i) −1407±122(ii) −5186±792	(i) 21.0(ii) 2.77	(i) 4.13 E5±0.00(ii) 2.54 E4±7.28 E3	(i) 2.42 E-6(ii) 3.94 × E-5	9.72
** Ca^2+^** ** Vs** ** Mg^2+^-bound CDD-C**	Sequential binding	(i) 1(ii) 1(iii) 1	(i) 1105±82.0(ii) −675.6±82.1(iii) −1429±31.7	(i) 30.4(ii) 22.8(iii) 10.2	(i) 6.75 E5±3.8 E5(ii) 3.05 E5±1.2 E5(iii) 1.85 E3±1.1 E2	(i) 1.48 E-6(ii) 3.28 E-6(iii) 5.41 E-4	13.79

### Surface plasmon resonance analysis

Binding studies for Caldendrin dimerization were carried out using the Biacore X-100 instrument and sensor chip CM5 (Biacore AB, GE Healthcare, Uppsala, Sweden) at 25°C as described previously [21]. Full-length Caldendrin or the N-terminus or C-terminus were coupled to the carboxymethylated dextran matrix of a sensor chip cell according to the manufacturers instructions. After equilibrating the sensor chip with HBS-P flow buffer (10 mM Hepes pH 7.4, 150 mM NaCl, 0.005% (v/v) Surfactant P20) at a flow rate of 10 µl/min, the dextran matrix was activated with a 7-minute pulse of 50 mM N-hydroxysuccinimide/200 mM N-ethyl-N′-(dimethylaminopropyl)- carbodiimide at a flow rate of 10 µl/min. Subsequently proteins were immobilized by injecting a 7-minute pulse of ligand solution. The excess of reactive groups on the chip surface was deactivated with a 7-minute pulse of 1 M ethanolamine hydrochloride pH 8.5, at a flow rate of 10 µl/min. For binding studies Caldendrin, the N- or -C-terminus were diluted at the indicated concentrations in the continuous flow buffer HBS-P containing defined Ca^2+^ and/or Mg^2+^-concentrations. Each analytic run was performed at 10 µl/min flow rate under the following conditions: 1 min equilibration of the chip with the indicated analysis buffer. Afterwards the analyte was injected in a 3-minute pulse (association time) followed by a 3-minute pulse with analysis buffer alone (dissociation time). Sequential sensorgrams were recorded at a flow rate of 10 µl/min. Controls were done with coupling of an unrelated GST-protein to the chip surface which resulted in no specific binding.

### Analytical gel-filtration

Homodimerization properties of Caldendrin were investigated by size exclusion chromatography performed on a ÄKTA FPLC system (ÄKTA purifier, GE Healthcare, UK). Untagged full length Caldendrin was applied on a superdex 75 column in Ca^2+^ -buffer (50 mM Tris, 100 mM KCl, 500 µM Mg^2+^ and 100 µM Ca^2+^), Ca^2+^-free buffer (50 mM Tris, 100 mM KCl, 500 µM Mg^2+^ and 200 µM EGTA) and Mg^2+^-buffer (50 mM Tris, 100 mM KCl, 500 µM and Mg^2+^). Eluted protein chromatograms were recorded at 214 nm. Eluted protein was processed for immunoblotting and the identity of the two peaks with Caldendrin was confirmed with a Caldendrin antibody.

### Dynamic light scattering

Dynamic light scattering (DLS) experiments were performed on filtered and ultracentrifuged samples of Caldendrin at 25°C using a Zetasizer Nano S instrument (Malvern Instruments). Untagged Caldendrin was buffer exchanged with 1x Tris-KCl buffer (Tris 50 mM, KCl 100 mM and Mg^2+^500 µM). The refractive index (1.337) and viscosity (0.8791) of 1x Tris-KCl was calculated and measurements were done with different protein concentrations with suitable attenuation power. The experiment was run in Ca^2+^ buffer (50 mM Tris, 100 mM KCl, 500 µM Mg^2+^ and 50 µM Ca^2+^) and Ca^2+^ free buffer (50 mM Tris, 100 mM KCl, 1 mM Mg^2+^ and 500 µM EGTA). Experiments were repeated several times to ensure accuracy.

### Förster Resonance Energy Transfer (FRET)

To test for intramolecular folding of Caldenrin FRET measurements were performed with HEK293T cell extracts as described previously [24]. Cells were transfected with mYFP-Caldendrin-mCFP, a mYFP-mCFP tandem construct as positive control or negative controls with mYFP and mCFP separately expressed. 48 h after transfection cells were harvested and lysed in TBS, pH 7.4; containing 1% Triton X-100 and protease inhibitor cocktail (EDTA-free Complete, Roche). Afterwards lysates were precleared by centrifugation at 13.000 rpm for 20 min at 4°C. Emission spectra were then measured using a fluorescence spectrophotometer (Model F-4500; Hitachi) with excitation at 425 nm (mCFP) and 485 (mYFP). The concentration of the mCFP, mYFP, or mCFP-mYFP fusion proteins in cell lysates was adjusted by measuring the YFP fluorescence.

## Results

Due to the highly basic N-terminus, low solubility and a strong tendency to form inclusion bodies when expressed in bacteria it turned out to be difficult to produce a full-length untagged Caldendrin protein for cation binding and structural studies. An intein-mediated purification was finally utilized and we succeeded to produce larger amounts of full-length protein (Figure S1A+B) with about 90–95% purity. Using this approach we were also able to produce myristoylated L- and S-CaBP1 with high purity at a scale of several milligrams (Figure S1A+B).

**Figure 1:**
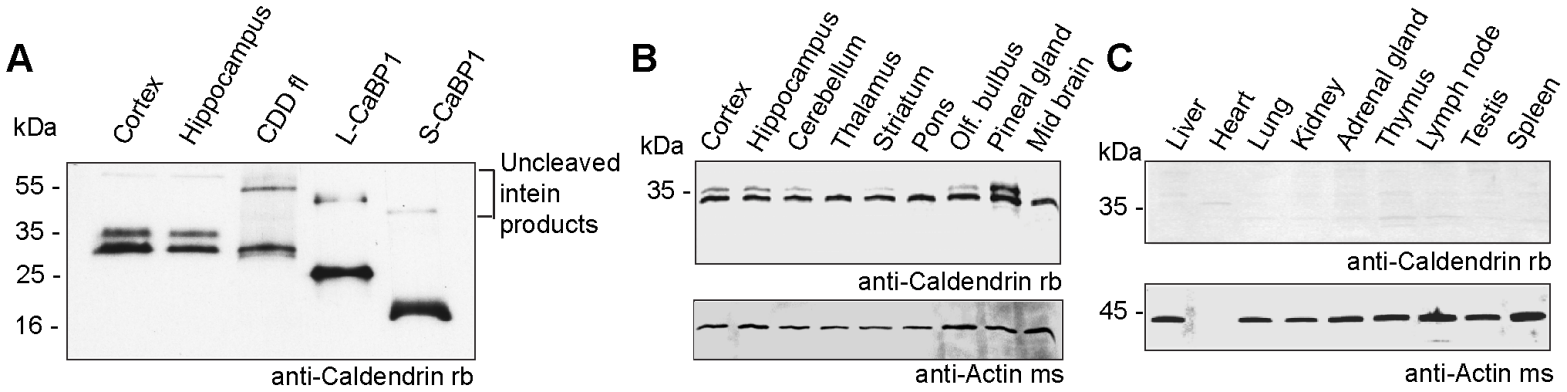
Expression anaylsis of Caldendrin, L- and S-CaBP1. (A) Immunoblot analysis reveals that bacterially expressed untagged Caldendrin migrates at 33 kDa like the smaller Caldendrin isoform in cortex and hippocampus of rat brain. Bacterially produced myristoylated L- and S-CaBP1 migrate at 25 kDa and 18 kDa respectively. Immunoreactivity is detected by anti-Caldendrin/CaBP1 rabbit antibody, directed against the common C-terminus of all three isoforms. 20 µg of brain samples are compared to ≈ 10 ng of purified proteins. The western blot shows Caldendin expression in different regions of rat brain (B) and in different rat organs (C). Caldendrin is detected by anti-Caldendrin/CaBP1 rabbit antibody (rb). Equal loading in all lanes was ensured by measuring the total protein concentraion (20 µg/lane) and verified with an anti-actin mouse antibody (ms). Note that consistant with previous reports the actin band is amost absent in heart tissue due to differnential expression of this marker.

In previous work we have described that Caldendrin is by far more abundant in brain than the other two splice isoforms [7], [8]. Subsequent conflicting reports indicated high levels of L- and to a lesser degree S-CaBP1 in forebrain regions [9]–[11]. We followed up on these conflicting results and loaded bacterially expressed and purified full-length Caldendrin, L-and S-CaBP1 on a SDS-page gel and then compared their migration behavior with endogenous protein expressed in brain using an antibody that is directed against the common C-terminus of all three splice isforms (Figure S1A/Figure 1A). We found that the antibody detects all three recombinant proteins at molecular weights of 33 kDa (Caldendrin), 25 kDa (L-CaBP1) and 19 kDa (S-CaBP1) (Figure 1A). In accord with previous data [5], [7], [12] we found a double band migrating at 33/36 kDa in cortex and hippocampus (Figure 1A). Most important only the double band at 33/36 kDa was present in total brain homogenates and even after long exposure times no bands appeared at 25 and 19 kDa (Figure 1A). The nature of this double band might be a differential posttranslational modification [5] or another splice isoform with an alternative open reading frame of 350 instead of 298 amino acids (NP_001028848.1).

We next analyzed with immunoblotting the expression of Caldendrin, L- and S-CaBP1 in different tissues and brain regions. In all brain regions examined we found the 33/36 kDa double band of Caldendrin while no bands appearing at 25 and 19 kDa were detected (Figure 1B). No expression of either isoform was detectable in other tissues than brain (Figure 1C). Collectively these experiments provide compelling evidence that Caldendrin is by far the most abundant isoform whereas L- and S-CaBP1 expression is not detectable in brain at the level of sensitivity that is provided by immunoblotting with the antibodies used.

**Figure 2:**
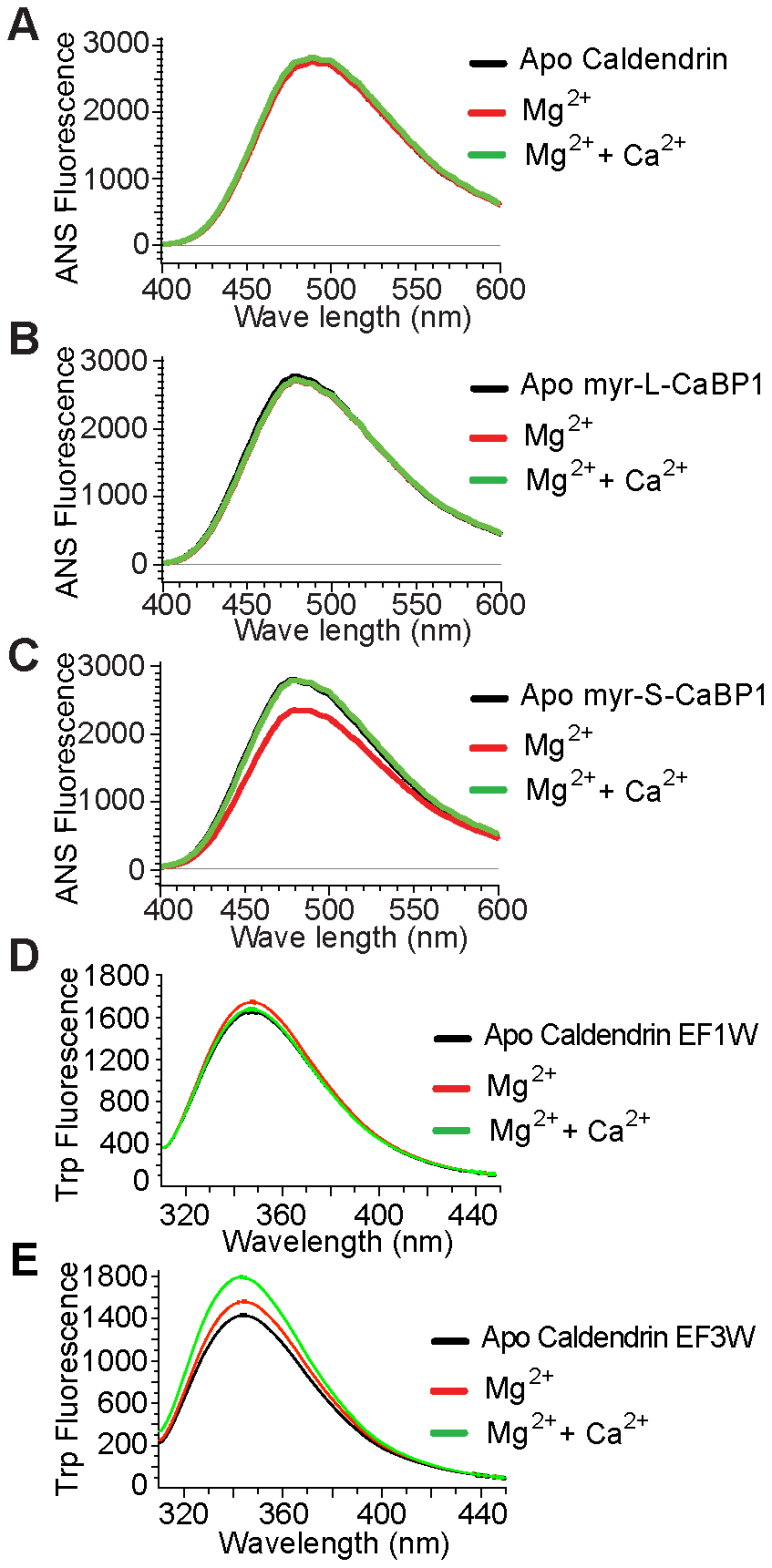
Extrinsic fluorescence ANS (1-Anilino-8-Naphthalin Sulfonate) fluorescence spectroscopy of Caldendrin and L- and S-CaBP1. (A) Mg^2+^ and Ca^2+^ titration to Mg^2+^ bound apo Caldendrin shows no change in the fluorescence intensity. (B) Titration of Mg^2+^ and Ca^2+^ titration to Mg^2+^ bound L-CaBP1 shows no change in the fluorescence intensity. (C) Effect of Mg^2+^ and Ca^2+^ on apo S-CaBP1. Titration with Mg^2+^ results in a significant decrease in the fluorescence intensity, whereas Ca^2+^ titration to Mg^2+^ bound S-CaBP1 reversed this effect. (D) Titration with Mg^2+^ affects tryptophane fluorescence intensity in EF1W mutant Caldendrin, (E) EF3W mutant Caldendrin exhibits large changes in tryptophane fluorescence intensity following Mg^2+^ and Ca^2+^ titration. For all titrations a protein concentration of 1 µM was used. The Mg^2+^ concentration was 0.5 mM and the Ca^2+^ concentration was 50 µM.

Most of the hitherto characterized Caldendrin/CaBP1 interactions with binding partners have been established with the common EF-hand domain containing C-terminus or the S-CaBP1 isoform. The impact of the basic N-terminal half of Caldendrin on the structure and ion binding properties of the common C-terminus are unknown. To learn more about the biophysical properties of Caldendrin we first performed steady-state fluorescence spectroscopy to test whether ion binding induces major structural changes that have impact on surface hydrophobicity. Since Caldendrin harbors no tryptophane we employed to this end fluorophore 8-anilino-1-naphthalene sulphonic acid (ANS) fluorescence spectroscopy. We saw that the overall changes in the spectra due to Mg^2+^/Ca^2+^ binding to Caldendrin and myristoylated L-CaBP1 were negligible (Figure 2A+B). However, we observed a robust change in surface hydrophobicity in Mg^2+^-bound S-CaBP1 (Figure 2C).

EF-hand Ca^2+^ sensors like Toponin-C and CaM undergo a close to open conformational change upon Ca^2+^ binding that exposes a hydrophobic surface which then can act as an interface for target recognition. Accordingly a close to open conformational change upon Ca^2+^ binding was observed in the NMR and crystal structures of the C-terminal EF-hand domain of S-CaBP1 [16]–[18]. To increase the sensitivity of the method we next generated mutant Caldendrin proteins having single Trp reporter groups in EF-hand 1 (F162W) and 3 (F239W). We indeed found a shift in steady state Trp-fluorescence in EF-hand 1 reporter mutants in the presence of Mg^2+^ (Figure 2D) and in EF-hand 3 reporter mutants in the presence of Ca^2+^ (Figure 2E), indicating a structural change of both EF-hand domains upon ion binding that has no impact on surface hydrophobicity.

**Figure 3:**
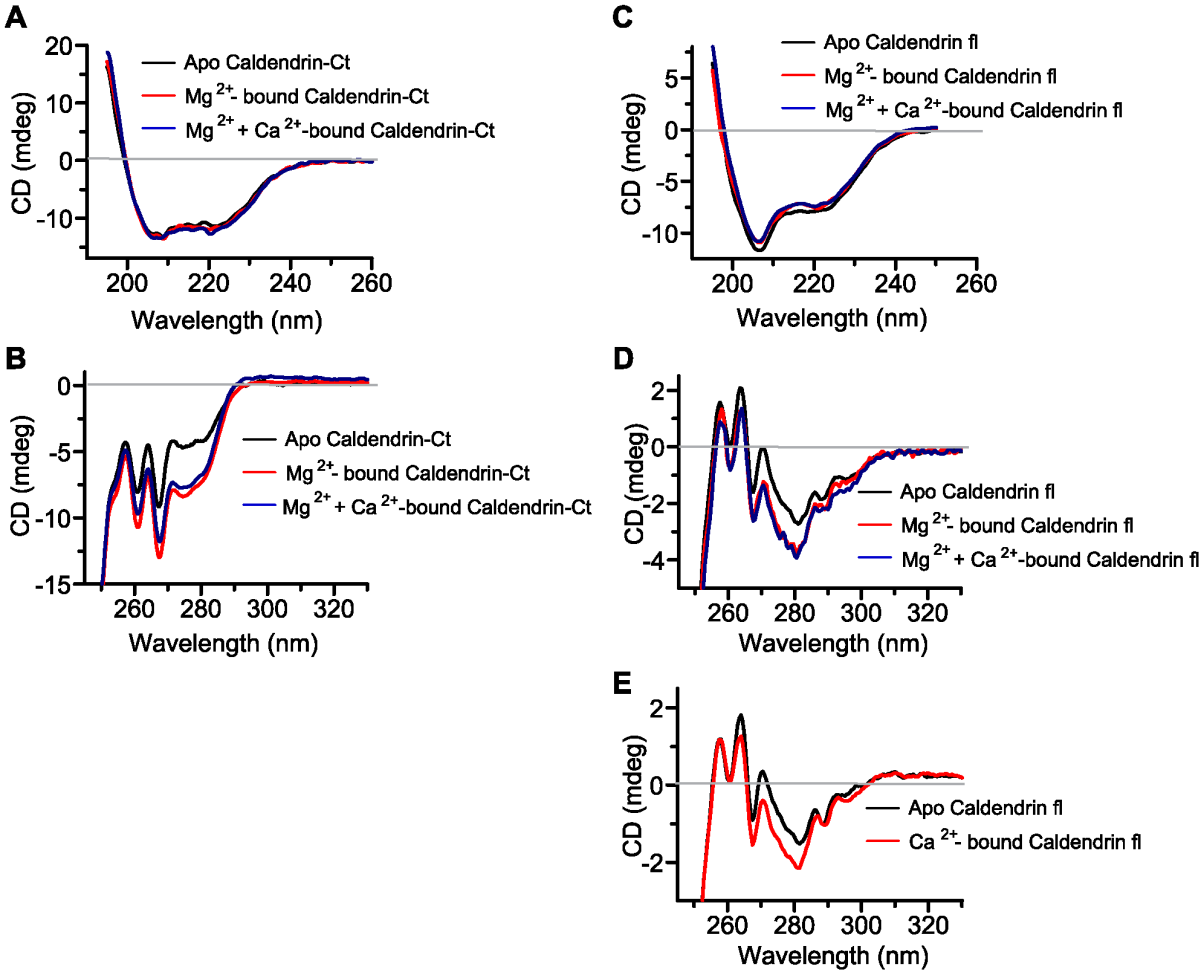
Far and Near UV- CD spectroscopy. (A) Far UV- CD spectra of the apo, Mg^2+^-bound and Mg^2+^+Ca^2+^ bound common C-terminus of Caldendrin/CaBP1 shows no conformational change in secondary structure. (B) Near UV- CD spectra of the apo, Mg^2+^-bound and Mg^2+^+Ca^2+^ bound common C-terminus of Caldendrin/CaBP1 exhibit significant change in tertiary structure. (C) Far UV- CD spectra of the apo-, Mg^2+^-bound and Mg^2+^+Ca^2+^-bound full length Caldendrin. Mg^2+^ binding to apo-Caldendrin causes a mild change in the secondary structure of the protein. Ca^2+^ binding to Mg^2+^- bound Caldendrin has no detectable effect on the secondary structure of the protein. (D) Near UV- CD spectra of apo-, Mg^2+^-bound and Ca^2+^-bound Mg^2+^+Ca^2+^-bound full length Caldendrin. Mg^2+^ binding to apo-CDD causes a significant change in the tertiary structure of the protein, whereas Ca^2+^ binding to Mg^2+^- bound Caldendrin has no effect. (E) Ca^2+^-titration of the apo-protein had a clear effect on the structure. Representative spectra for each condition are shown.

**Figure 4:**
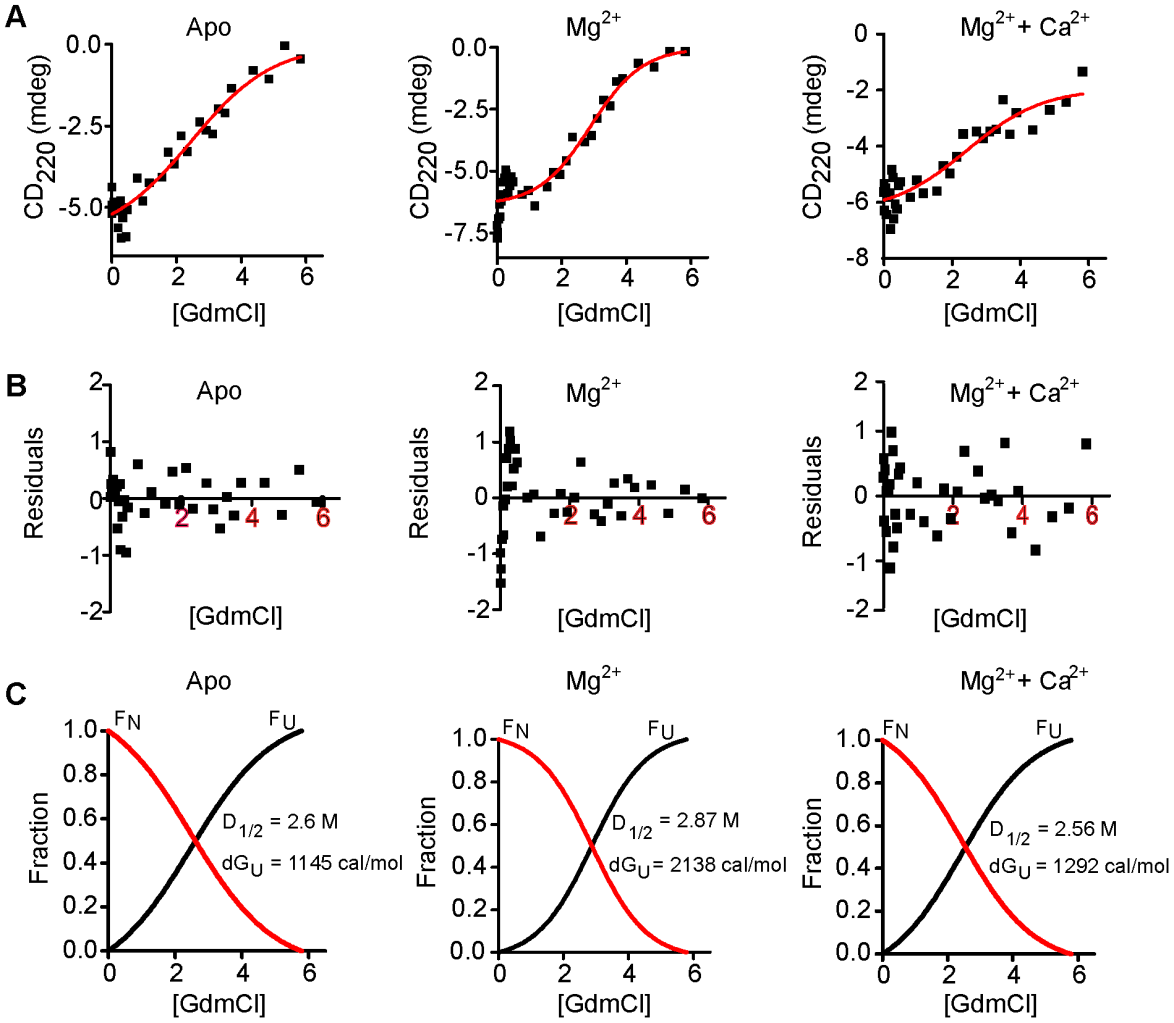
Equilibrium chemical unfolding. Equilibrium chemical unfolding of full length Caldendrin with guanidinium hydrochloride (GdmCl) in the absence of any ligand (Apo-CDDfl), in presence of 5 mM MgCl_2_ and 1 mM EGTA (Mg^2+^-CDDfl); and 5 mM MgCl_2_ and 1 mM CaCl_2_ (Mg^2+^Ca^2+^- CDDfl). The upper lane shows (A) the ellipticity values of plotted against [GdmCl] (black squares) and the fitting obtained (black curve) using a two-state unfolding model. The middle lane (B) shows the quality of model fitting in terms of residuals and the bottom lane (C) shows the normalized fits for fraction folded (FN; red curve) and fraction unfolded (FU; black curve) along with the standard free energy change of unfolding (ΔGU) obtained.

To corroborate these findings we next performed Far- and Near-UV CD spectroscopy to monitor conformational changes upon ligand binding (Figure 3). Far-UV (195–250 nm) CD spectra showed that Caldendrin has a high α-helical content, which does not change significantly upon the addition of Mg^2+^ or Ca^2+^ (Figure 3C). Similar results were obtained with the common C-terminus (Figure S1A; Figure 3A). These data are in accordance with observations made in S-CaBP1, which also does not show major changes in global secondary structure upon Ca^2+^-binding [19], [22]. The purified proteins had a properly folded tertiary structure as evident from the distinct peaks of phenylalanine and tyrosine in the Near-UV CD spectra (Figure 3B+D). Titrating the apo-protein with Mg^2+^ caused large changes in the spectra indicating significant structural changes in the protein upon ligand binding (Figure 3B+D). Ca^2+^-titration of the apo-protein also had an effect on the structure (Figure 3E). Surprisingly, when Ca^2+^ was titrated to Mg^2+^-bound Caldendrin no change was induced in the spectrum under these conditions (Figure 3D), indicating only a minor structural change, which is probably not detectable in Near-UV CD spectroscopy owing to the reduced sensitivity of this technique in the absence of a Trp residue. We next determined equilibrium chemical unfolding monitored by Far-UV CD using GdmCl. A two-state model of unfolding gave the best fit in all conditions tested and the free energy change of unfolding (ΔGU) thereby obtained, was used to draw conclusions. From this analysis, it was found that Mg^2+^-binding enhanced the stability of the apo-protein (Figure 4A–C), whereas Ca^2+^-binding reduced the structural stability of Mg^2+^-bound Caldendrin.

**Figure 5:**
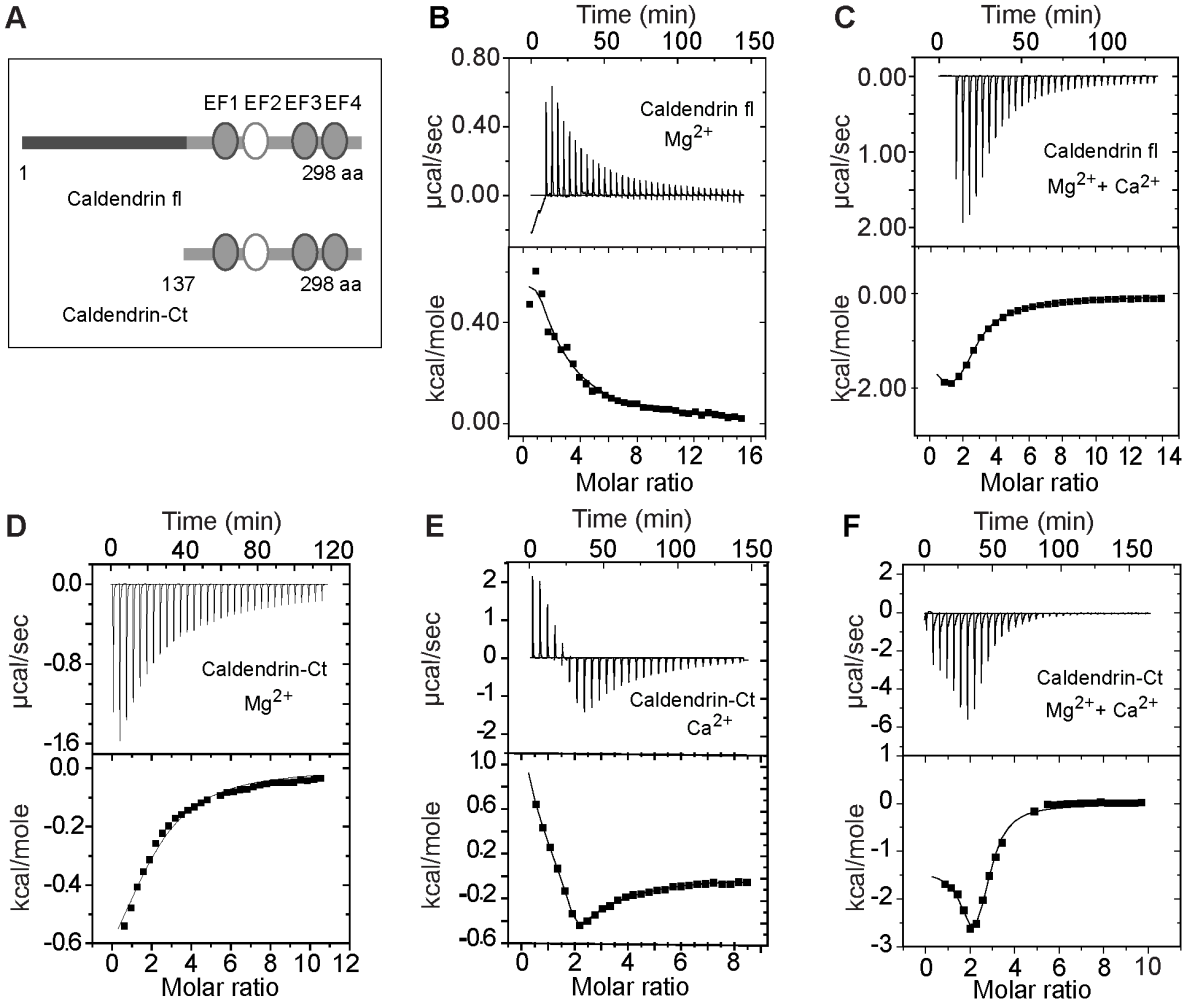
ITC analysis of Caldendrin and the common C-terminus of Caldendrin, L- and S-CaBP1. (A) Cartoon showing the Caldendrin (CDD) full length (1–298 residues) and Caldendrin Ct (137–298 residues) proteins used for ITC. (B) ITC of Apo-Caldendrin with Mg^2+^. Titration was carried out with 10 mM MgCl_2_. Protein concentration was 43 µM. Data were fitted using a sequential binding model. (C) Ca^2+^ titration to Mg^2+^ saturated Caldendrin. Titration was carried out with 10 mM CaCl_2._ Data were fitted using a sequential binding model. (D–F) ITC analysis of Mg^2+^, Ca^2+^ and Ca^2+^ titration to the Mg^2+^ bound common C-terminus of Caldendrin/CaBP1 (173 µM). The upper part in each in each panel shows the traces of calorimetric ion titration and the lower panel represents the integrated binding isotherms obtained from various best fit models. This figure is connected to Table 1 which includes the corresponding ITC values.

Taken together the CD data suggest that Mg^2+^ has strong impact on the global conformation of Caldendrin whereas Ca^2+-^binding to Mg^2+^-liganted protein has much less impact. To learn more about the affinity and stoichiometry of Ca^2+^- and Mg^2+^-binding to Caldendrin we then performed ITC. Intracellular free Mg^2+^ concentrations are in the range of 1 mM and many neuronal calcium sensor proteins show Mg^2+^ binding including S-CaBP1 (see above and [19]). Therefore we first performed ITC experiments with Mg^2+^ titration followed by Ca^2+^ titration. These experiments revealed that in the presence of Mg^2+^, Caldendrin has a comparable global dissociation constant for Ca^2^-binding (Figure 5A+C/Kd = 10.9 µM/Table 1) as S-CaBP1 (Kd = 7 µM/Table 1; [18]). This affinity is lower than those reported for other NCS proteins and also Calneurons [2]. Interestingly, the binding of Mg^2+^ to Caldendrin is an endothermic process, which is in contrast to myristoylated S-CaBP1 where Mg^2+^ binding is an exothermic process (Figure 5B) [19]. Calculation of a one-site model results in an affinity of 75µM, which is 4-times higher than the Mg^2+^ binding affinity of S-CaBP1 (Table 1/[19]). Strikingly we found a dissociation constant for Mg^2+^ binding to apo-Caldendrin of 2.4 µM when we calculated a model of sequential binding for one high and one low affinity binding site (Table 1). The error margins for both models were found to be in an acceptable range. However, titration of the N-terminus alone (20 µM of protein with up to 10 mM Mg^2+^) resulted in no specific binding (data not shown). Thus, if a second low-affinity binding site exists it might be either located in the second EF-hand domain of Caldendrin or is based on the coordination of Mg^2+^ by amino acids from the N and C terminus.

Taken together the data suggest that the presence of the N-terminus appears to influence the mechanism of Mg^2+^-coordination at least of EF-hand 1 [19]. To confirm this striking difference we performed ITC experiments with the common C-terminus of Caldendrin/CaBP1 (residues 137–298). These experiments showed similar Mg^2+^- and Ca^2+^-binding affinities to those reported for S-CaBP1 (Figure 5A+D–F/K_d_ for Mg^2+^227 µM; K_d_ for Ca^2+^9.7 µM for the Apo-Protein and 13.7 µM for the Mg^2+^-bound C-terminus).

**Figure 6:**
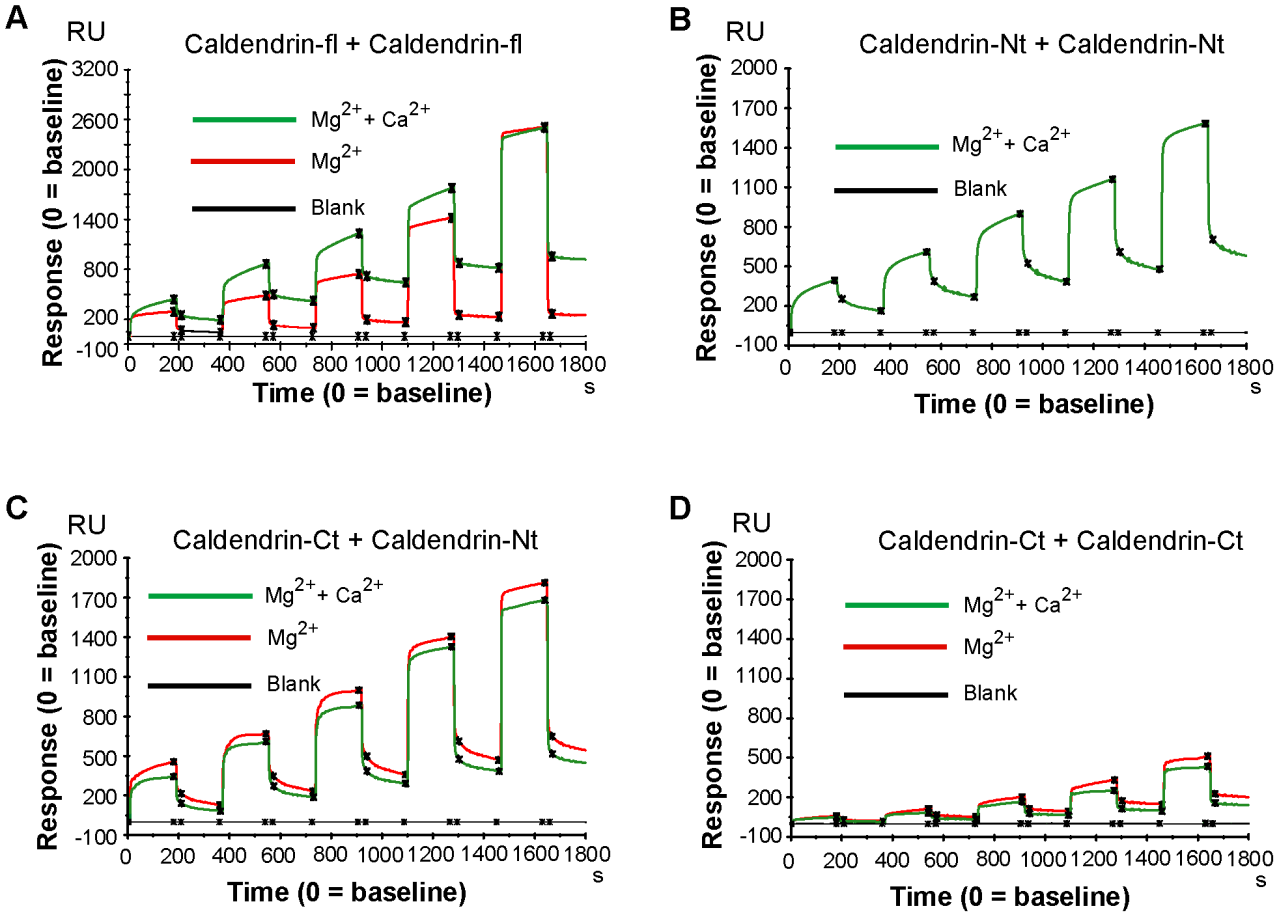
Surface plasmon resonance analysis. (A) Untagged full length Caldendrin was immobilized on a CM5 surface and the full-length Caldendrin was also injected as analyte. (B) The N-terminus of Caldendrin was immobilized on a CM5 surface and also injected as analyte in running buffer. (C) The common C-terminus (CDD-Ct) was immobilized on the sensor chip and the N-terminus injected as analyte. (D) The common C-terminus (CDD-Ct) was immobilized on the sensor chip and also injected as the analyte. (A–D) The running buffer always contained 50 mM Tris-Cl and 100 mM KCl with 1 mM Mg^2+^/1 mM EGTA (red) or 1 mM Mg^2+^/500 µM Ca^2+^ (green). Amount of protein in the running buffer was 5, 10, 20, 40, and 80 µg (increasing protein levels correspond to increasing amplitudes). RU: response units.

Collectively these results make it plausible that the basic N-terminus of Caldendrin, which is lacking in S-CaBP1, might physically associate and interact with the acidic C-terminus and could thereby influence Mg^2+^-binding. To prove whether the N-terminus can directly interact with the C-terminus we immobilized the common C-terminus (residues 137–298) on the surface of a sensor chip via amide coupling for subsequent surface plasmon resonance (SPR) analysis (Figure 6A). We found saturable binding with high molar binding activity deduced from the dissociation phase of the interaction when we injected an N-terminal peptide (residues 1–136) as the analyte (Figure 6A/Table 2). The binding was reduced in the presence of 0.5 mM Ca^2+^/1 mM Mg^2+^ as compared to 1 mM Mg^2+^/1 mM EGTA (Figure 6A). We have previously reported dimer formation between the EF-hand containing C-terminus of Caldendrin/CaBP1 [28]. Similar results were obtained in SPR analysis where we found a direct interaction of the C-terminus (residues 137–298) with a modest decrease in binding in the presence of 0.5 mM Ca^2+^ (Figure 6B/Table 2). In addition, we measured high response units and molar binding activity when we coupled the full-length protein to the sensor chip and injected full-length protein as analyte (Figure 6C/Table 2). We next tested for self-association of the N-terminus of Caldendrin and found a tight association with molar binding activities comparable to those observed for the interaction of the N-terminus with the C-terminus (Figure 6D/Table 2). Thus, the intermolecular interaction of both N-termini is as strong as the intramolecular interaction of the N- with the C-terminus and dimerization of the common C-terminus.

**Table 2:** Molar binding activity for different dimer combinations in the presence of Ca^2+^ and EGTA.

immobilized + injectant	Ca^2+^	EGTA
CDD fl + CDD fl	7,57×10^−2^	2,15×10^−2^
CDD-Nt + CDD-Nt	2,10×10^−1^	1,92×10^−1^
CDD-Ct + CDD-Nt	1,63×10^−1^	2,03×10^−1^
CDD-Ct + CDD-Ct	3,73×10^−2^	5,41×10^−2^

We next used *in vitro* FRET of YFP-Caldendrin-CFP in HEK293T cell extracts to corroborate these findings. Following heterologous expression we saw a strong FRET signal of the full-length Caldendrin fusion protein that was tagged with YFP at the N- and CFP at the C-terminus (Figure S2). No FRET signal was obsered when Caldendrin tagged with YFP at the N-terminus was coexpressed with Caldendrin fused with CFP at the C-terminus (Figure S2). This suggests that also the protein expressed in eukaryotic cells might exhibit preferential backfolding of the N-terminus as compared to formation of an anti-parallel dimer (Figure S2).

**Figure 7:**
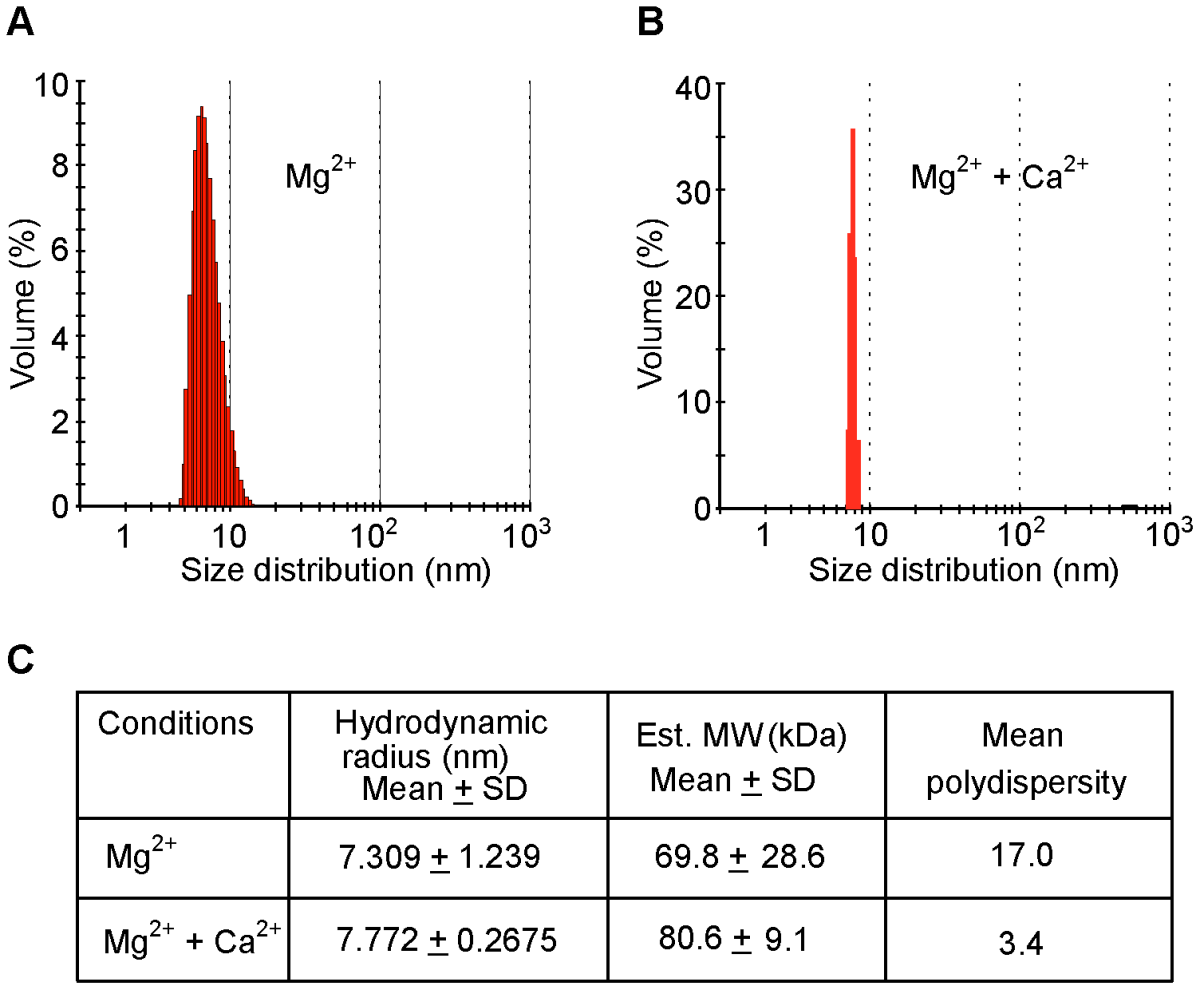
Dynamic light scattering (DLS) experiments further demonstrate the existence of a Caldendrin homodimer. (A) The radius of Caldendrin in the presence of Mg^2+^. Polydispersity suggests that Mg^2+^-bound Caldendrin may exhibit conformational heterogeneity or exist as an equilibrium mixture of monomer and dimer species. (B) DLS shows that Caldendrin in the Ca^2+^-bound state preferentially forms a dimer. (C) Table showing the molecular mass of Caldendrin, polydispersity in the presence of Mg^2+^ (1 mM Mg^2+/^1 mM EGTA) or Mg^2+^+Ca^2+^ (1 mM Mg^2+^/50 µM Ca^2+^). Experiments were repeated with three different purifications and from each puriifcation samples were measured six to seven times.

We next assessed dimerization of full length Caldendrin with size exclusion chromatography. Intein-purified untagged full length Caldendrin saturated with Mg^2+^ was applied on a superdex 75 column. Eluted protein chromatograms (at 214 nm) displayed two peaks at the size of a dimer and monomer (Figure S3). To test if the presence of Ca^2+^ has an effect on transitions between different states of Caldendrin self-association, dynamic light scattering (DLS) experiments that allow an estimatimation of the hydrodynamic radius and oligomerization state of the protein in solution were performed. The theoretical mass of the bacterially expressed Caldendrin full length protein is 33.08 kDa. The DLS size histograms showed estimated molecular weights of 69.8 and 80.6 kDa with poly-dispersities of 17 and 3.4% for Mg^2+^- and Ca^2+^-bound Caldendrin, respectively, suggesting a lower level heterogeneous population of protein species (Figure 7A–C). The polydispersity suggests that Mg^2+^-bound Caldendrin may exhibit conformational heterogeneity whereas the protein in the Ca^2+^-bound state shows a more prominent dimer population (Figure 7A–C).

## Discussion

Several studies have shown that CaBP1 might play a role in regulating [Ca^2+^]_i_ levels in neurons via an interaction with inositol 1,4,5-triphosphate receptors and various VGCC [9]–[11], [16], [25]–[31]. In this paper we show that Caldendrin is by far the most abundant isoform deriving from the Caldendrin/CaBP1 gene in rat brain, whereas the shorter isoforms L- and S-CaBP1 are barely detectable in all brain regions examined. Of note, while this paper was under review a similar expression pattern of Caldendrin and L- and S-CaBP1 was reported in mouse brain [32].

Caldendrin has a key role in targeting the synapto-nuclear messenger Jacob to synaptic sites in a Ca^2+^-dependent manner [20] but very little is known about other targets. Most of the hitherto characterized Caldendrin/CaBP1 interactions with binding partners have been established *in vitro* or in heterologous systems with the common C-terminus or S-CaBP1. Importantly, it has been shown previously that the modes of binding and regulation of L-type CaV1.2 channel are not identical for Caldendrin and S-CaBP1 [11]. To learn about the physiological role and significance of previously reported interactions it will be necessary to verify whether reported binding partners of the common C-terminus in brain are also bound by Caldendrin with a similar functional outcome.

This is of particular relevance in light of the absence of a major change in surface hydrophobicity of Caldendrin upon Ca^2+^-binding of Mg^2+^-liganted protein. The phyisological free Mg^2+^-concentration in brain makes it plausible that caldendrin is always in a Mg^2+^-bound state. Interestingly, this change is also lacking in L-CaBP1 and both isoforms differ from S-CaBP1 only in their distinct N-terminal domains. Many Ca^2+^ sensors bind to their targets in an ON-OFF manner. In their Ca^2+^-bound (ON) form they associate with their targets, whereas in their apo-form (OFF), they dissociate from them; the exposure of the interfacial hydrophobic surface controls this switch. An enhancement of surface hydrophobicity upon Ca^2+^-binding was observed in the current and previous studies for S-CaBP1 [16], [18]. Taken together the data point towards a different role of Ca^2+^ for target interactions of Caldendrin as opposed to the classical switch-like role. Several interactions of the common C-terminus are Ca^2+^-independent and conceivable is therefore a Ca^2+^-independent pre-association with a functional outcome that is then modulated by Ca^2+^. In conjunction with low Ca^2+^-binding affinity of Caldendrin and the high abundance of CaM, a binding mode that cannot be competed by other Ca^2+^-sensors is likely and it is an interesting question whether a bound target might alter the Ca^2+^-binding dynamics of Caldendrin with respect to affinity as well as on/off rates for Ca^2+^.

A surprising finding was the high affinity of Caldendrin for Mg^2+^. Mg^2+^-binding causes a conformational change in Caldendrin and surprisingly Ca^2+^- titration of the Mg^2+^-bound protein had no further impact on the structure. In near-UV CD experiments, we found that Mg^2+^-binding alters the structure of Caldendrin and equilibrium chemical unfolding experiments revealed that Mg^2+^-binding increases the structural stability of Caldendrin, indicating a structural role of Mg^2+^ for protein folding. It is possible that folding back of the N-terminal domain of Caldendrin impacts on Mg^2+^-binding, and that Caldendrin might therefore behave different from S-CaBP1 and neuronal Ca^2+^-sensors in this regard. The first EF-hand domain will always be Mg^2+^-bound, which excludes interdomain cooperativity in Ca^2+^-binding as known for other CaM-like four EF-hand Ca^2+^-sensor proteins. The rigid first EF-hand domain and folding back of the N-terminus might enable unique Ca^2+^-dependent target association. Of interest in this regard is the fact that Caldendrin contains several PxxP motifs in the N-terminal domain. It is conceivable that SH3 domain binding could influence the folding back of the N-terminus and thereby Mg^2+^-binding affinitiy and at the same time stabilizes a Ca^2+^-independent interaction of the C-terminus that is then subsequently modulated by Ca^2+^-binding of the second EF-hand domain. Alternatively, folding back of the N-terminus appears to be modulated by Ca^2+^ and the PxxP motifs might be more accessible in Ca^2+^-bound Caldendrin.

Besides this intramolecular interaction, we also found evidence for intermolecular interactions of the N- and C-terminus. Structural studies carried out on S-CaBP1 have yielded variable results with respect to its dimer status [16], [18], [19], which could be due to differences in experimental conditions. In this study, Caldendrin was found to form dimers in the absence of Ca^2+^ in biochemical as well as biophysical experiments. Binding of Ca^2+^ has a modulatory influence on self-association and folding back of the N-terminus. Collectively the data suggest that Caldendrin in a Ca^2+^ free state might be a dimer via self-association of the N-terminus, whereas binding of Ca^2+^ facilitates dimerization of the EF-hand domain containing part and loosens the association of the N- with the C-terminus. Intra- and intermolecular interactions of the N-terminus might happen in parallel. Thus both, folding back and dimerization appear not to be mutually exclusive and several intermediate states are conceivable. Along these lines it is tempting to speculate that dimerization, folding back and Ca^2+^-binding might affect the interface for protein-protein interactions.

## Conclusion

Caldendrin exhibits complex self-association, high Mg^2+^- and low Ca^2+^-binding affinity and little conformational change upon titration with Ca^2+^, indicating a different mode of target binding than other neuronal Ca^2+^-sensors.

## Supporting Information

Figure S1 **Scheme of Caldendrin/CaBP1 protein organization, production and purification.** (A) Purification of Caldendrin, L- and S-CaBP1 by the Impact Intein purification system. Coomassie staining shows Caldendrin full-length at different steps of purification starting from soluble protein in the supernatant (SN), flow through (FT), matrix bound (MB), eluted to dialyzed and concentrated protein. Myristoylated protein purification by the Impact purification system (right panel). Molecular masses are indicated.
(TIF)

Figure S2 **Caldendrin adopts a back-folded conformation:** ***in vitro*** **FRET analysis.** (A) Fluorescence spectra showing increased FRET efficiency in the positive control (CFP-YFP) and no FRET is observed in the negative control (CFP+YFP), where CFP and YFP were expressed by different vectors. (B) A clear FRET signal was detected in the YFP channel when YFP was fused to the N- and CFP to the C-terminus of Caldendrin (YFP-CDD-CFP). (C) Co-transfection of Caldendrin fused with YFP- to the N-terminus and Caldendrin fused with CFP to the C-terminus resulted in no FRET signal in the YFP-channel.
(TIF)

Figure S3 **Size exclusion chromatography.** (A) Overlaid gel filtration chromatograms of Caldendrin injected in the presence of Mg^2+^ (500 µM Mg^2+^ red 200 µM EGTA; red) or Mg^2+^+Ca^2+^ (500 µM Mg^2+^/100 µM Ca^2+^; black). Chromatograms of Caldendrin were compared with low molecular weight calibration kit proteins (GE Healthcare/green). The first peak (10–11 elution volume in ml) eluted at the size of the dimer and the second peak (11.5–12 ml) eluted at the size of the monomer. Absorption of all chromatograms was done at the wavelength 214 and 280 nm and with the 500 µl fractions size. (B) The chromatogram shows that recombinant Caldendrin elutes at the size of a monomer (11–11.5 ml) in the presence of 0.02% sodium dodecyl sulfate (SDS/black). A Caldendrin dimer was prominent when we incubated the protein with 0.01% Glutaraldehyde (GA) (red). Chromatograms of Caldendrin were compared with low molecular weight calibration kit proteins (GE Healthcare/green).
(TIF)
