# Supplementary figures and images for: Molecular Dynamics of the Neuronal EF-Hand Ca2+-Sensor Caldendrin
Source: PLoS One. 2014 Jul 24;9(7):e103186. doi: 10.1371/journal.pone.0103186 (PMC4110014; doi:10.1371/journal.pone.0103186)

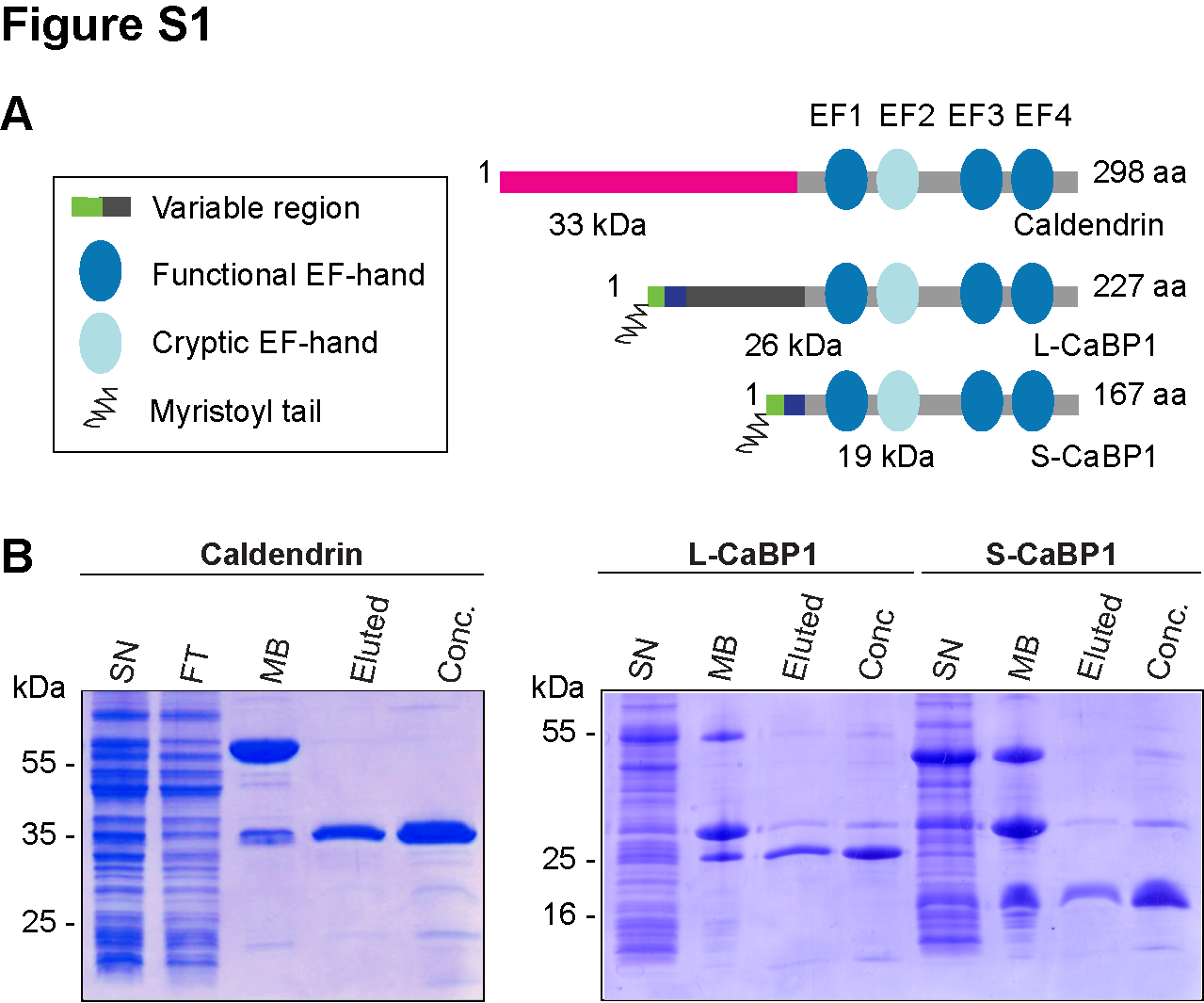

Supplement: Figure S1 — Scheme of Caldendrin/CaBP1 protein organization, production and purification. (A) Purification of Caldendrin, L- and S-CaBP1 by the Impact Intein purification system. Coomassie staining shows Caldendrin full-length at different steps of purification starting from soluble protein in the supernatant (SN), flow through (FT), matrix bound (MB), eluted to dialyzed and concentrated protein. Myristoylated protein purification by the Impact purification system (right panel). Molecular masses are indicated. (TIF) [file pone.0103186.s001.tif]

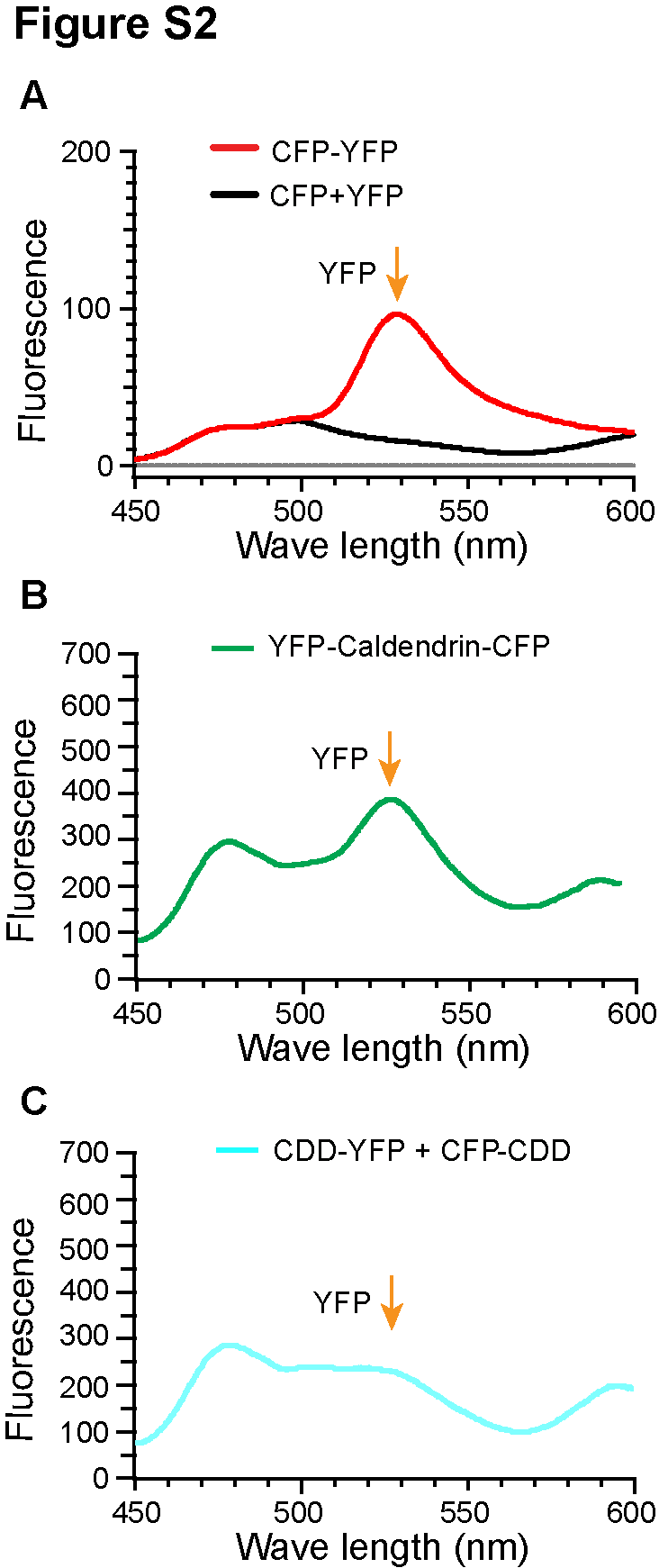

Supplement: Figure S2 — Caldendrin adopts a back-folded conformation: in vitro FRET analysis. (A) Fluorescence spectra showing increased FRET efficiency in the positive control (CFP-YFP) and no FRET is observed in the negative control (CFP+YFP), where CFP and YFP were expressed by different vectors. (B) A clear FRET signal was detected in the YFP channel when YFP was fused to the N- and CFP to the C-terminus of Caldendrin (YFP-CDD-CFP). (C) Co-transfection of Caldendrin fused with YFP- to the N-terminus and Caldendrin fused with CFP to the C-terminus resulted in no FRET signal in the YFP-channel. (TIF) [file pone.0103186.s002.tif]

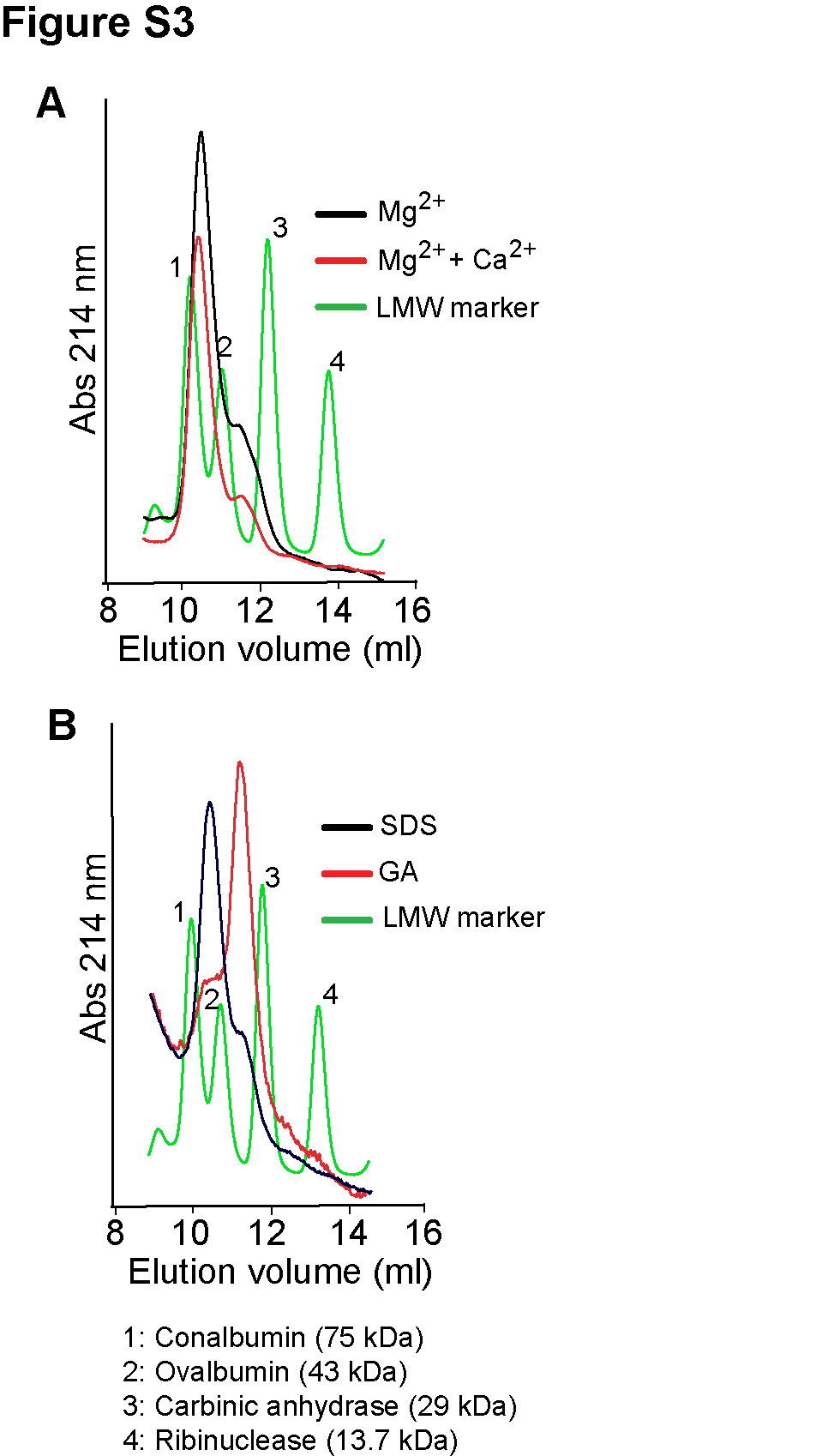

Supplement: Figure S3 — Size exclusion chromatography. (A) Overlaid gel filtration chromatograms of Caldendrin injected in the presence of Mg2+ (500 µM Mg2+ red 200 µM EGTA; red) or Mg2++Ca2+ (500 µM Mg2+/100 µM Ca2+; black). Chromatograms of Caldendrin were compared with low molecular weight calibration kit proteins (GE Healthcare/green). The first peak (10–11 elution volume in ml) eluted at the size of the dimer and the second peak (11.5–12 ml) eluted at the size of the monomer. Absorption of all chromatograms was done at the wavelength 214 and 280 nm and with the 500 µl fractions size. (B) The chromatogram shows that recombinant Caldendrin elutes at the size of a monomer (11–11.5 ml) in the presence of 0.02% sodium dodecyl sulfate (SDS/black). A Caldendrin dimer was prominent when we incubated the protein with 0.01% Glutaraldehyde (GA) (red). Chromatograms of Caldendrin were compared with low molecular weight calibration kit proteins (GE Healthcare/green). (TIF) [file pone.0103186.s003.tif]
